# Allelic Expression Imbalance of *JAK2* V617F Mutation in *BCR-ABL* Negative Myeloproliferative Neoplasms

**DOI:** 10.1371/journal.pone.0052518

**Published:** 2013-01-22

**Authors:** Hye-Ran Kim, Hyun-Jung Choi, Yeo-Kyeoung Kim, Hyeoung-Joon Kim, Jong-Hee Shin, Soon-Pal Suh, Dong-Wook Ryang, Myung-Geun Shin

**Affiliations:** 1 Brain Korea 21 Project, Center for Biomedical Human Resources, Chonnam National University, Gwangju, South Korea; 2 Departments of Laboratory Medicine, Chonnam National University Medical School and Chonnam National University Hwasun Hospital, Hwasun, Korea; 3 Department of Hematology and Genome Research Center for Hematopoietic Disease, Chonnam National University Medical School and Chonnam National University Hwasun Hospital, Hwasun, Korea; University of Porto, Portugal

## Abstract

The discovery of a single point mutation in the *JAK*2 gene in patients with *BCR/ABL*-negative myeloproliferative neoplasms (MPNs) has not only brought new insights and pathogenesis, but also has made the diagnosis of MPNs much easier. Although, to date, several mechanisms for the contribution of single *JAK*2V617F point mutation to phenotypic diversity of MPNs have been suggested in multiple studies, but it is not clear how a unique mutation can cause the phenotypic diversity of MPNs. In this study, our results show that allelic expression imbalance of *JAK*2 V617F mutant frequently occurs and contributes to phenotypic diversity of *BCR-ABL*-negative MPNs. The proportion of *JAK*2 V617F mutant allele was significantly augmented in RNA levels as compared with genomic DNA differently by distinct MPNs subtypes. In detail, preferential expression of *JAK2* mutant allele showed threefold increase from the cDNA compared with the genomic DNA from patients with essential thrombocythemia and twofold increase in polycythemia vera. In conclusion, allelic expression imbalance of *JAK*2 V617F mutant proposes another plausible mechanism for the contribution of single *JAK*2 point mutation to phenotypic diversity of MPNs.

## Introduction

Myeloproliferative neoplasms (MPNs) are clonal stem cell disorders encompassing a very heterogeneous complex of different entities which are defined by distinct clinical and cytomorphological phenotypes and, in some instances, known genetic features. They are characterized by increased and effective proliferation of one to three hematopoietic cell lineages in the bone marrow associated to increased peripheral blood parameters [1]. According to recently revised 2008 World Health Organization (WHO) classification of hematological neoplasms [2], MPNs are subclassified into eight separate entities: chronic myelogenous leukemia, polycythemia vera (PV), essential thrombocythemia (ET), primary myelofibrosis (PMF), systemic mastocytosis, chronic eosinophilic leukemia not otherwise specified, chronic neutrophilic leukemia, and unclassifiable MPN. Their phenotypic diversity is attributed to different configurations of abnormal signal transduction, resulting from a spectrum of mutations affecting protein tyrosine kinases or related molecules. Therefore, histology-based classification and diagnostic criteria for these disorders can be refined by employing molecular disease markers [2].

The tyrosine kinase Janus kinase 2 (*JAK*2) is directly linked to the pathogenesis of MPN with the identification of *JAK*2 V617F as a recurring gain-of function mutation [3]. The *JAK2* V617F point mutation is now widely used as a diagnostic marker for *BCR/ABL*-negative MPNs. The human *JAK*2 gene is located on chromosome 9p24 and was first cloned in 1989 from the cDNA library of a murine hematopoietic cell line. Like Janus, usually pictured with two faces, *JAK*2 has two important domains: JH1 and JH2. JH1, located near the carboxyl terminus of the protein, is the active kinase domain and possesses the tyrosine residues, which are phosphorylated when *JAK*2 is activated. JH2, a kinase-like domain, lacks actual kinase activity. Rather, JH2 appears to exert an inhibitory effect on JH1. The V617F mutation lies in the auto-inhibitory JH2 domain, and therefore increases *JAK*2 kinase activity, confers cytokine-independent growth on cell lines, and is associated with erythropoietin independent growth of primary cells [4].

With almost all PV cases, and roughly 50% of patients with ET and PMF, carry a *JAK*2 V617F mutation localized on chromosome 9p24 [3]. *JAK*2 V617F mutation occurs at one allele (the heterozygous pattern) or both alleles (the homozygous pattern) in PV patients, and previous studies have reported that the *JAK*2 V617F homozygous group presents higher hemoglobin concentration, higher incidence of myelofibrotic change than the *JAK*2 V617F heterozygous group. Among MPNs patients with the *JAK*2 mutation, the ratio of *JAK*2 V617F mutant allele to *JAK*2 total allele (V617F mutant allele plus wild type *JAK*2 allele) is suggested to involve the clinical phenotypes of MPNs [5]. In addition, a thorough examination of the *JAK2* gene in *JAK2* V617F-negative PV patients led to the identification of various *JAK2* mutations in exon 12. Especially, mutation in *JAK2* exon 12 has so far been found only in PV but the rarity of this mutation makes it difficult to predict whether other MPN entities could also harbor this type of *JAK2* mutation at lower frequencies [6].

To date, the explanation for the phenotypic diversity in MPN involves several hypothesis including: *JAK*2 V617F mutation in a different progenitor cells than the primitive stem cells, occurrence of a second acquired genetic event, or dosage of expression in *JAK*2 mutation [7]. But it still remains puzzling how a unique mutation and lower proportion of *JAK*2 mutants in genomic DNA can be involved in development and diversity of diseases, and also how the frequency of the *JAK*2 V617F mutation in MPNs differs according to methods and research groups. To address these issues we investigated the frequency, status, and allelic variation of *JAK*2 V617F mutation in Korean MPN patients and demonstrated the usefulness of *JAK*2 V617F mutation as a molecular marker for treatment response and/or disease progression in *BCR-ABL*-negative MPNs using quantitative real time polymerase chain reaction (qPCR) and pyrosequencing.

## Results

### Clinical and laboratory features

Brief laboratory and clinical features of 78 MPN patients at diagnosis were presented in Table 1. MPN patients with *JAK2*V617F mutation disclosed higher white blood cell count and hemoglobin concentration than those without *JAK* V617F mutation. But *JAK* V617F mutation-positive patients had lower platelet count than mutation-negative patients (Table S1).

**Table 1:** Demographic and hematologic characteristics of 78 patients with MPNs.

Characteristics	PV	ET	PMF	UC
Number	26	42	7	3
Median age (range)	57 (33–81)	54 (19–80)	61 (49–74)	63 (57–68)
Male/Female (No.)	12/14	25/17	4/3	2/1
WBC (×10^3^/uL [median, range])	14 (6–26)	10 (5–24)	16 (5–29)	15 (6–24)
PLT (×10^3^/uL [median, range]]	525 (161–940)	1,066 (375–2513)	322 (23–615)	707 (430–921)
Hb (g/dL [median, range])	16.5 (8.8–21.4)	13.4 (7.4–17.8)	9.5 (7–12.2)	11.0 (7.7–11.6)

PV, polycythemia vera; ET, essential thrombocythemia; PMF, primary myelofibrosis; UC, unclassifiable MPN; WBC, white blood cell count; PLT, platelet count.

### Frequency of JAK2 V617F mutation in Korean MPN patients

The *JAK*2 V617F mutation was detected using AS-PCR, REA, direct sequencing (Figure S1 and Figure S2) and pyrosequencing. Each molecular method disclosed a marked difference of detection rate: REA 30.8%, direct sequencing 46.2%, pyrosequencing 48.7%, qPCR 62.8% and AS-PCR 70.5% (Table 2). The *JAK*2 V617F mutation was present in over half of Korean patients with *BCR-ABL*-negative MPNs, giving an overall frequency of 56.4%. A single point *JAK*2 V617F mutation was identified in 23 (88.5%) of 26 patients with PV, 24 (57.1%) of 42 with ET, six (85.7%) of seven with MF and two (66.7%) of three with unclassifiable MPN detected by at least one method with positive result (Table 2).

**Table 2:** Frequency of *JAK*2 V617F mutation in Korean MPNs patients.

Methods	MPNs (No. of patients)					Total No. (%)
	Results	PV (26)	ET (42)	PMF (7)	UC (3)	78
Direct sequencing	Mutant	8	2	3	0	13
	Mixed	10	10	3	0	23
	Wild-type	8	30	1	3	42
	No (%)	18 (69.2)	12 (28.6)	6 (85.7)	0 (0)	36 (46.2)
REA	Mutant	7	2	3	1	13
	Mixed	4	4	3	0	11
	Wild-type	15	36	1	2	54
	No (%)	11 (42.3)	6 (14.3)	6 (86)	1 (33.3)	24 (30.8)
PSQ	Mixed	18	11	7	2	38
	Wild-type	8	31	0	1	40
	No (%)	18 (69.2)	11 (26.2)	7 (100)	2 (66.7)	38 (48.7)
qPCR	Mixed	24	17	6	2	49
	Wild-type	2	25	1	1	29
	No (%)	24 (92.3)	17 (40.5)	6(85.7)	2 (66.7)	49 (62.8)
AS- PCR	Mixed	23	24	6	2	55
	Wild-type	3	18	1	1	23
	No (%)	23 (88.5)	24 (57.1)	6 (85.7)	2 (66.7)	55 (70.5)
Overall*	No (%)	23 (88.5)	25 (59.5)	7 (100)	2 (66.7)	57 (73.1)

REA, restriction enzyme-based assay; PSQ, pyrosequencing; AS-PCR, allele-specific PCR with DPO primer; other Abbreviations as in Table 1.

* , calculated from the number of patients having at least one method with positive result.

### Determination of JAK2 V617F mutant allele burden

Real-time quantitative PCR (qPCR) and pyrosequencing were used for the semi-quantitative determination of *JAK*2 V617F mutation load. The linear regression relating the two methods is shown in Figure S3. The correlation coefficient (r), r-square value (r^2^), slope and intercept were 0.863, 0.744, 1.023 and −0.423, respectively, indicating a fair correlation between the two methods (Figure S3). Patients with PMF showed the highest proportion of *JAK*2 V617F mutant alleles among the three diseases. PV patients had higher values than those with ET. Ranges of the proportion of mutant alleles in patients with PV, ET, and PMF were 2–92% (57.5±28.2, mean±SD), 1–88% (39.1±31.2) and 72–90% (71.7±31.5), respectively. These differences between the diseases studied were statistically significant (*P = 0.006*) in the non-parametric Kruskall-Wallis test (Table 3).

**Table 3:** Quantitation of *JAK*2 mutant by real time PCR and pyrosequencing from the patients with JAK2 V617F mutation.

MPNs with JAK2 V617F mutation	*JAK2* V617F mutant (%)		
		qPCR^1^	PSQ
PV (n = 23)	Mean^2^	58.4	58.2
	SD	16.2	25.1
ET (n = 25)	Mean^2^	39.1	37.7
	SD	21.6	27.8
PMF (n = 7)	Mean^2^	71.7	74.3
	SD	11.5	8.7

1 Formula for proportion of *JAK*2 mutant: 1/(1+1/2ΔCt), ΔCt = Ct wild type – Ct mutant.

2 Statistically significant difference among each MPNs by Kruskall-Wallis test (*P = 0.006*).

qPCR, real-time PCR; other Abbreviations as in Table 1.

### Homozygous and heterozygous JAK2 V617F mutation

Homozygous *JAK*2 V617F mutation was defined as more than 50% of *JAK*2 mutant allele. Heterozygous *JAK*2 V617F mutation was mainly observed in ET patients, which was further confirmed using TA cloning analysis (Figure S4). In contrast, all PMF patients and the majority of PV patients harbored homozygous *JAK*2 V617F mutation. The frequency of homozygous among patients with MF, PV, and ET showed a statistically significant difference (*P = 0.021*).

### Allelic expression imbalance of JAK2 V617F mutation

**Figure 1:**
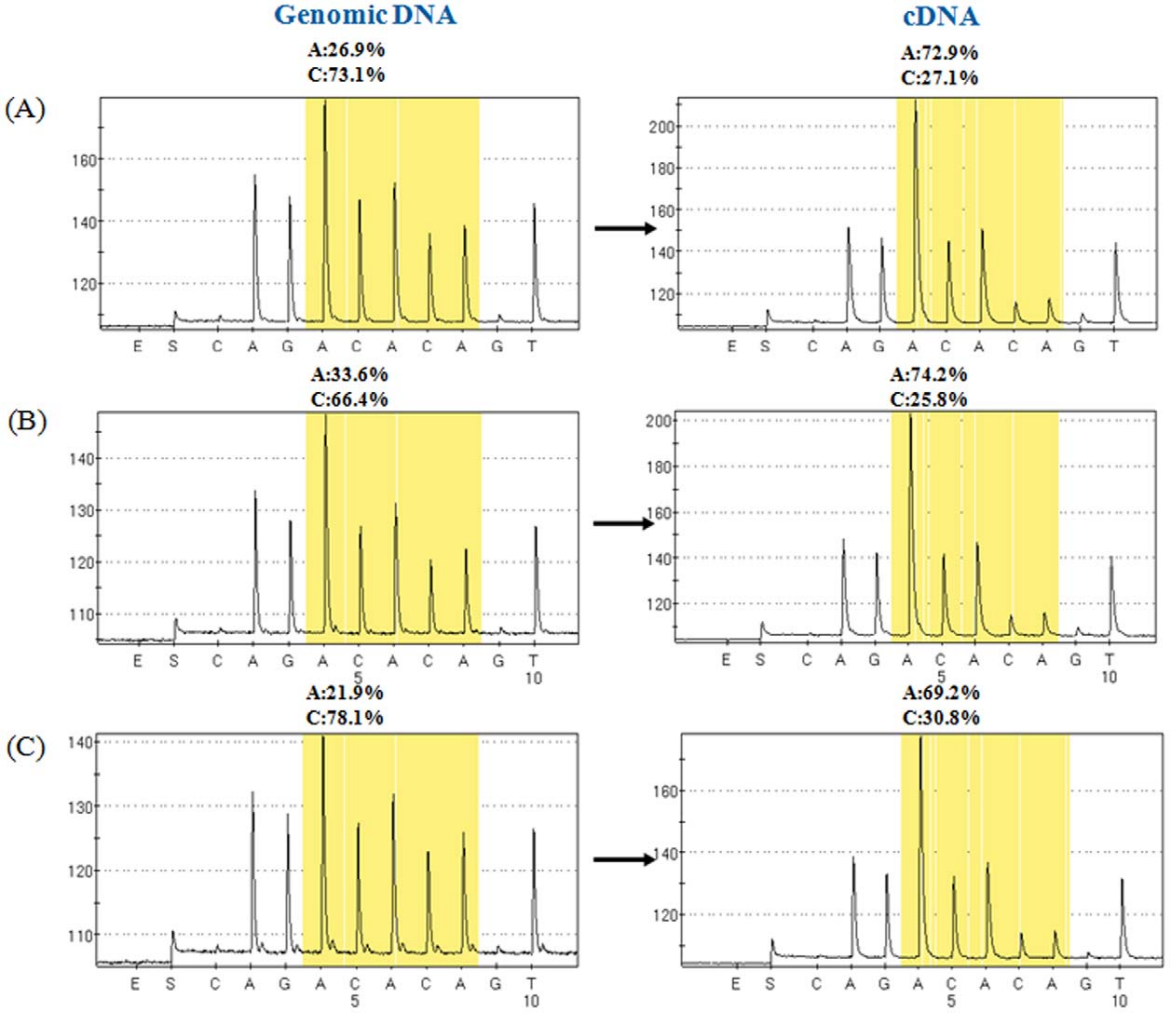
Allelic expression imbalance of *JAK*2 V617F mutation. Pyrosequencing assay for the detection of *JAK*2 1849G>T mutation was applied in genomic DNA and corresponding cDNA samples from patients with ET (A) and PV (B and C). Reversed pyrogram disclosed allelic expression imbalance (amplification in cDNA) of the *JAK*2 mutant.

**Figure 2:**
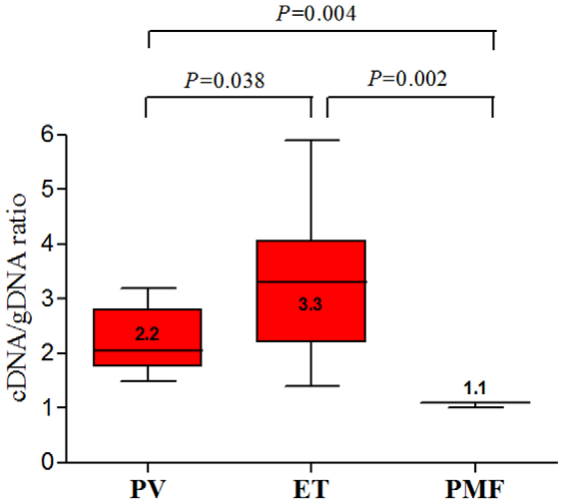
Ratio of the *JAK*2 mutant allelic load of gDNA and cDNA. Amplification of *JAK*2 mutant allele was observed in all MPN patients. The ratio of *JAK*2 mutant from genomic and cDNA disclosed markedly difference from MPN patients (n = 57) with the *JAK*2 V617F mutation. Allelic expression imbalance of *JAK*2 V617F mutation is associated with MPN phenotypes.

To determine whether *JAK*2 V617F mutant and endogenous wild type *JAK*2 alleles were differentially expressed according to RNA and DNA level, we checked them from each of total RNA (cDNA) and genomic DNA (gDNA) samples from the same patients. The proportion of *JAK*2 V617F mutant allele was significantly augmented at RNA levels rather than gDNA. Preferential expression of *JAK2* mutant allele showed a 3-fold increase from the cDNA compared with the gDNA from patients with ET and 2-fold increase in PV (Figure 1). The ratio (mean±SD) of cDNA versus gDNA for *JAK*2 V617F mutant allele in ET, PV and PMF patients was 3.3±1.4, 2.2±0.6 and 1.1±0.04, respectively (Table S3, Figure 2). Thus, allelic expression imbalance of *JAK2* V617F mutation was common in patients with MPNs.

### JAK2 V617F mutant allele burden and therapeutic interventions

**Figure 3:**
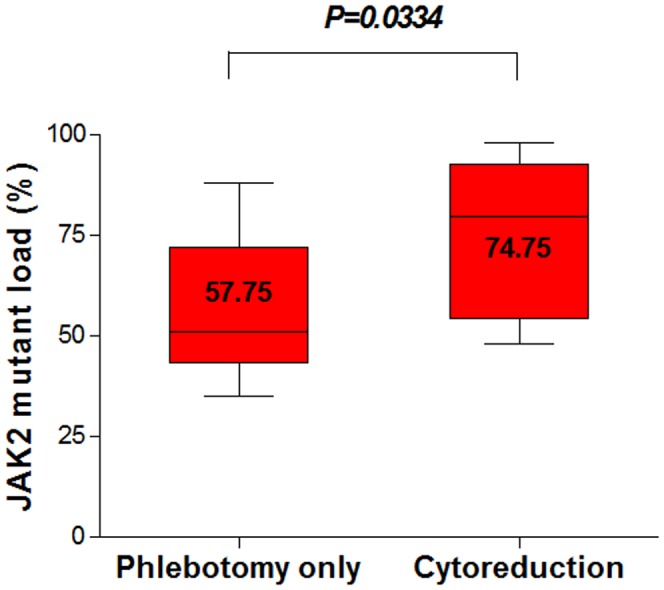
Proportion of the *JAK*2 V617F mutant according to treatment response in 15 PV patients with the *JAK*2 V617F mutation. *JAK*2 mutant load predicted treatment response and strategy at diagnosis.

Of the total patients, in 15 patients with PV, *JAK*2 mutation status was sequentially evaluated at three time points during the clinical course with treatment. Patients receiving cytoreduction chemotherapy had a higher proportion of *JAK*2 mutant alleles than patients who were treated with phlebotomy only (Mann Whitney test, P<0.05) (Figure 3).

## Discussion

There are several suggested mechanisms for the contribution of single *JAK*2 point mutation to phenotypic diversity of MPNs. However, how a single amino-acid substitution in *JAK2* gene can result in three different phenotypes still remains unclear [7]. The current study was investigated to search for another plausible mechanism for addressing this issue. In this study, allelic expression imbalance of *JAK*2 V617F mutant often occurred and the degree of it was distinctly different according to *BCR-ABL*-negative MPNs subtypes. Together with discovering these novel findings, *JAK*2 V617F mutant allele burden was implicated in therapeutic interventions and there was difference of detection rate for a single point *JAK*2 V617F mutation by each of molecular methods such as AS-PCR, REA, direct sequencing and pyrosequencing.

To date, several hypotheses for mechanism regarding how a single mutation can be contributed to different MPN subtypes are under investigation such as differences in the targeted hematopoietic stem cells, host modifier polymorphisms, intensity of *JAK2* V617F signaling, presence of other somatic mutations, or the presence of a pre-*JAK2* event that may vary according to the MPN phenotype [8]. Although multiple studies have provided some evidence for and against each hypothesis, it now seems that none of them is the absolute right one, but in fact all of them are [8]. The present study documents that the proportion of *JAK*2 V617F mutant allele was significantly augmented in RNA levels especially in patients with ET and PV. In detail, preferential expression of *JAK2* mutant allele showed threefold increase from the cDNA compared with the genomic DNA from patients with ET and twofold increase in PV. Recent case study reports that cDNA quantitative analysis revealed high *JAK2* mRNA levels with >96% *JAK2*V617F, implying an mRNA expression almost 100-fold higher for recombined alleles in *V617F/V617F* cells than for alleles in *WT/WT* cells [9]. Our novel findings of the current study correspond well with the results from this case report.

The variations in genetic expression at the transcript level account for a major part of phenotypic variations not only within and among species but etiology and phenotypic variability of complex diseases [10], [11]. Individuals heterozygous for *cis*-acting polymorphisms (or epigenetic modification of specific alleles) that affect gene expression or mRNA processing show a different level of mRNA expression originating from one allele compared with the other. This is called allelic expression imbalance, which can serve as an integrative quantitative measure of any and all *cis*-acting factors [10]. Allelic expression imbalance has been used to identify genes influenced by cis-acting regulatory SNPs and the measurement of allelic expression has shown considerable promise for quantitative analysis of cis-acting factors that determine gene expression and mRNA processing [11]. Allelic expression imbalance is measured by determining the number of genomic DNA molecules for each allele in comparison to the number of allelic mRNA molecules in the target tissue [10]. In this study, our results show that allelic expression imbalance of *JAK*2 V617F mutant frequently occurred and associated with phenotypic diversity of *BCR-ABL*-negative MPNs. However, this finding was a kind of observational result, so further study was needed to have the power to show a correlation.

Whereas evidence of higher JAK2 V617F allele burden in PV than ET have been reported in previous studies [12], data on allelic burden in PMF remains to be validated. Six out of seven PMF patients were post-PV PMF (n = 4) and post-ET PMF 9 (n = 2). Therefore, most of our PMF patients are secondary PMF, which may result in harboring the highest proportion of JAK2 V617F mutant load. The JAK2 V617F mutant load was higher in PMF>PV>ET subtypes, together with the allelic expression imbalance showing higher in the ET>PV>PMF patients in this study. For addressing this issue, we assumed that if the loading of JAK2 V617F mutant already reached to homozygous status (relatively high proportion of mutant allele) in genomic DNA level, the possibility of augmentation for JAK2 V617F loading in RNA level may no longer occur.

Changes in *JAK*2 V617F allele burden and clinical manifestation including prognostic relevance are tightly associated in most instances [12]. The current study also investigated the type of therapeutic interventions and therapy responses according to *JAK*2 V617F mutant burden at diagnosis. PV patients receiving treatment with a phlebotomy only had a relatively low proportion (58.9±20.1%) of the *JAK*2 mutant at diagnosis, while patients (n = 9) with additional cytoreduction chemotherapy had a higher proportion of *JAK*2 mutant (76.6±19.0% p = 0.04185). This premature finding suggests that *JAK*2 mutant allele burden might play a role to determine the type of therapeutic interventions as well as prognosis. The results also suggest that *JAK*2 mutation might be used as a new marker for disease progression and minimal residual disease detection in *BCR-ABL*-negative MPNs using qPCR and/or pyrosequencing.

Recently several sensitive methods for the detection of *JAK*2 V617F mutation have been published, most are based on qPCR [13]. Although a number of comparative studies have been performed, that describe the sensitivity and specificity of various techniques, Taqman-based qPCR assays were known to be the best techniques to quantify *JAK2*V617F [14]. We early experienced significant false positive results when conventional AS-PCR method was introduced for the detection of *JAK2* V617F mutation. To avoid pitfalls observed in conventional AS-PCR, the DPO-based AS-PCR system was used, which generate high PCR specificity (Figure S5) [15]. In this study, detection rate of AS-PCR with DPO primers was high than other molecular methods for detection of *JAK2* V617F, especially in patients with ET.

The frequency of *JAK*2 V617F mutation in Korean ET and PV patients was lower than those of previous reports from Western countries [3], [16]. The *JAK*2 V617F mutation was present in over half of Korean patients with *BCR-ABL*-negative MPNs, giving an overall frequency of 56.4%. Prevalence of the *JAK*2 V617F mutation varies in independent research groups and different populations [17]. These differences could be attributed to ethnic variation and different molecular methods but needs confirmation larger population studies for addressing racial difference of prevalence. Similar to previous reports, MPN patients with the *JAK*2 V617F mutation showed a higher white blood cell count and hemoglobin concentration, and lower platelet count than those without the *JAK*2 V617F mutation in our study [18].

In conclusion, allelic expression imbalance of *JAK*2 V617F mutant was frequently occurred in patients with *BCR-ABL*-negative MPNs, which contributed to phenotypic diversity of MPNs. Therefore allelic imbalance in the gene expression of *JAK*2 V617F mutant could provide the underlying mechanisms to elucidate phenotypic diversity of *BCR-ABL*-negative MPNs.

## Materials and Methods

### Patients

A total of 78 patients with *BCR-ABL*-negative MPNs comprising 42 cases of ET, 26 PV, seven PMF and three unclassifiable MPN were enrolled in the present study. The diagnosis of PV, ET and PMF was made according to 2008 World Health Organization criteria. All patients were examined at the time of diagnosis without receiving prior treatment. Mononuclear cells from bone marrow were separated by density gradient centrifugation using ficoll hypaque (sigma-aldrich, St. Louis, USA) and washed twice in phosphate-buffered saline. Genomic DNA and total RNA were extracted using an AccuPrep Genomic DNA extraction kit and ExiPrep cell total RNA kit (Bioneer, Daejeon, Korea), respectively. This study was approved by the Institutional Review Board of Chonnam National University Hwasun Hospital, Hwasun, Korea. Written informed consent was provided by all the patients.

### Allele specific PCR with dual-priming oligonucleotide (DPO) primers

For the detection of *JAK*2 V617F mutation, the current study employed allele-specific PCR (AS-PCR) with a novel dual-priming oligonucleotide (DPO) primers (Seeplex™ *JAK2* genotyping kit; Seegene, Seoul, Korea) [15]. The DPO primer system differs from conventional primer systems, which is composed by two separate priming regions joined by a polydeoxyinosine linker (poly (I) linker) (Table S2). Deoxyinosine has a relatively low melting temperature compared to the natural bases, due to weaker hydrogen bonding so that the poly (I) linker will form a bubble-like structure at a certain annealing temperature, which itself is not involved in priming and separates a single primer into a two functional regions (Figure S5). After Poly(I) linker activation at relatively low melting temperature, the longer 5′-segment preferentially binds to the template DNA and initiates “stable annealing”. And then, the short 3′-segment selectively binds to a target site and determines target-specific extension. According to manufacturer guideline, initial denaturation at 94°C for 5 min, 35 cycles of 94°C for 30 sec, 60°C for 1.5 min, 72°C for 1.5 min with a final extension at 72°C for 5 min were carried out by a thermal cycler (Applied Biosystems, Foster City, CA).

### Restriction enzyme-based assay

Published primers and PCR condition were used for restriction enzyme-based assay (REA) [4]. Amplified 460 bp fragments were digested with *Bsa*XI (New England Biolabs, Hitchin, UK) for 4 h at 37°C, and then analyzed on a 1.8% agarose gel. The mutant allele remained undigested whereas the wild-type allele was digested into 241 bp, 189 bp, and 30 bp fragments.

### Direct sequencing

The reaction mixture for amplified product contained 1 µM of each primer, 0.2 mM of each deoxynucleoside triphosphate. 5 µL of PCR 10× buffer and 2.5 U *Taq* DNA polymerase (TaKaRa, Shiga, Japan) (Table S2). The samples were amplified in a TaKaRa PCR thermal cycler (TaKaRa) by using the following cycling parameters: one initial cycle of 1 min at 96°C, followed by 35cycle of 30 s at 94°C, 50 s at 58°C, and 1 min at 72°C, with one final cycle of 5 min at 72°C. PCR products were directly sequenced in both directions on ABI 3100 analyzer by BigDye chemistry (Applied Biosystems, Foster City, CA, USA). We analyzed sequencing traces both manually and with Mutation Surveyor (version 2.0; Softgenetics, State College, PA, USA).

### Real time PCR for quantification of JAK2 V617F mutant

Quantitative *JAK*2 V617F mutant alleles was performed as previously described using highly sensitive real-time PCR. Briefly, two real-time qPCR reactions were performed in parallel with a common forward primer and Taqman probe and only differing in the use of a reverse primer specific for the JAK2 wildtype and the V617F mutated DNA respectively [19]. The nucleotide sequence of each primer was provided in Table S2.

### Pyrosequencing for quantification of JAK2 V617F mutant

A published protocol [20] was used for sample preparation and pyrosequencing for *JAK*2 V617F genotyping and allele quantitation. After PCR, the biotinylated strand was captured on streptavidin Sepharose beads (Amersham Biosciences; Uppsala, Sweden) and annealed with the sequencing primers for the strand (Table S2). Pyrosequencing was performed separately for the strands using PSQ HS 96 Gold SNP Reagents and the PSQ HS 96 pyrosequencing machine (Biotage, Uppsala, Sweden).

### Assessment of allelic expression of JAK2 mutation

Allelic expression of *JAK*2 mutation was assessed using total RNA and DNA samples from the same MPN patients. Prior to reverse transcription, total RNA samples were first treated with RNase-free DNase I, and the RNA samples were then reverse-transcribed into first strand cDNA with 1 µg of total RNA, 4 µL of 5× first strand buffer, 4 µL of 0.1 mM dithiothreitol, 1 µL of 500 µg/mL random primer (Promega; Madison, WI, USA), 4 ml of 10 mM dNTP mixture and 200 U of SuperScript II RNase H2 reverse transcriptase (Life Technologies; Rockville, MD,USA). The reaction was incubated at 42°C for 60 min. Reverse transcription reactions were always carried out in the presence or absence of reverse transcriptase to ensure that genomic DNA did not contaminate the subsequent PCR. The quantization of *JAK*2 V617F mutant in cDNA and genomic DNA was performed using qPCR and pyrosequencing.

## Supporting Information

Table S1 **Clinical and laboratory features according to** ***JAK*** **2 V617F mutation status.**
(DOCX)

Table S2 **Primer sequences of allele specific PCR, direct sequencing, pyrosequencing and real time PCR for the detection of** ***JAK*** **2 V617F mutation.**
(DOCX)

Table S3 **Ratio of cDNA to gDNA of the** ***JAK*** **2 mutant allelic load in Korean MPN patients.**
(DOCX)

Figure S1 **Results of allele-specific PCR with a novel dual-priming oligonucleotide (DPO) system.**
(DOC)

Figure S2 **Detection of** ***JAK*** **2 mutation using direct sequencing (A) and REA (B).** A: Reverse chromatogram showed wild-type sequence and G to T mutation in *JAK*2 wild-type pattern in a patient with PMF (left panel). Mixed and mutant sequences in two patients with PV (center and right panel). Arrows indicate the relevant base. B: *JAK*2 *Bsa*XI digestion were used to genotype DNA from unfractionated peripheral blood leucocytes from patients with ET.
(DOCX)

Figure S3 **Correlation study between quantitative PCR (qPCR) and pyrosequencing (PSQ).** Regression analysis of qPCR vs PSQ molecular methods for quantitative determination of JAK2 mutant in the bone marrow cells at diagnosis. Two methods showed good agreement with 0.863 and 0.744 of the correlation coefficient (r) and r-square value (r^2^), respectively.
(DOCX)

Figure S4 **TA cloning analysis for further confirmation of heterozygote** ***JAK*** **2 1849G>T mutation.** Direct sequencing chromatogram showed mixtures of *JAK*2 wild (1849G) and mutant allele (1849T).
(DOCX)

Figure S5 **Scheme of the allele-specific DPO primers for the detection of** ***JAK*** **2 V617F mutation.** Internal control (813 bp) of PCR was used *JAK*2-F primer and *JAK*2-R primer. *JAK2*-F primer and *JAK*2-wr primer was used for detection of *JAK*2 wild type (617V, 534 bp). *JAK*2-R primer and *JAK*2-mf primer was used for detection of *JAK*2 mutant type (617F, 352 bp).
(DOCX)
