# Supplementary material for: Allelic Expression Imbalance of JAK2 V617F Mutation in BCR-ABL Negative Myeloproliferative Neoplasms
Source: PLoS One. 2013 Jan 22;8(1):e52518. doi: 10.1371/journal.pone.0052518 (PMC3551963; doi:10.1371/journal.pone.0052518)
Supplement: Table S1 — Clinical and laboratory features according to JAK 2 V617F mutation status. (DOCX) [file pone.0052518.s001.docx]

**Table S1. Clinical and laboratory features according to *JAK*2 V617F mutation status**

| **Demographics** | ***JAK*2 V617F** | ***JAK*2 wild-type** |
| --- | --- | --- |
| No (%) | 57 (73) | 21 (27) |
| Male No (%) | 27 (47) | 13 (62) |
| Female No (%) | 30 (53) | 8 (38) |
| Mean age (range) | 56.8 (19~81) | 51.7 (34~80) |
| WBC [x10^3^/uL (median, range)] | 14.68 (5.04~29.4)* | 10.9(4.8~62)* |
| PLT [x10^3^/uL (median, range)] | 660 (161~1612) | 965(181~2513) |
| Hb [g/dL (median, range)] | 14.3 (7~21.4) | 13.7(7.4~19.4) |

*, statistically significance, P<0.05.
