# Supplementary material for: Allelic Expression Imbalance of JAK2 V617F Mutation in BCR-ABL Negative Myeloproliferative Neoplasms
Source: PLoS One. 2013 Jan 22;8(1):e52518. doi: 10.1371/journal.pone.0052518 (PMC3551963; doi:10.1371/journal.pone.0052518)
Supplement: Table S2 — Primer sequences of allele specific PCR, direct sequencing, pyrosequencing and real time PCR for the detection of JAK 2 V617F mutation. (DOCX) [file pone.0052518.s002.docx]

**Table S2. Primer sequences of allele specific PCR, direct sequencing, pyrosequencing and real time PCR for the detection of *JAK*2 V617F mutation**

| **Methods** | **Primers** | **Sequences (5' to 3')** |
| --- | --- | --- |
| Direct sequencing | F | 5'-GGGTTTCCTCAGAACGTTGA-3' |
|  | R | 5'-TCATTGCTTTCCTTTTTCACAA-3' |
| Pyrosequencing | F | 5'-GAAGCAGCAAGTATGATGAGCA-3' |
|  | R | 5'-TGCTCTGAGAAAGGCATTAGAAA-3' |
|  | SQ | 5'-TTACTTACTCTCGTCTCCAC-3' |
| Real time PCR [1[9](#_ENREF_9)] | F | 5'-CTTTCTTTGAAGCAGCAAGTATGA-3' |
|  | Wild-R  Mutation-R | 5'-GTAGTTTTACTTACTCTCGTCTCCACAtAC-3'  5'-GTAGTTTTACTTACTCTCGTCTCCACAtAA-3' |
|  | Probe | 5'-FAM-TGAGCAAGCTTTCTCACAAGCATTTGGTTT-TAMRA-3' |

F, forward primer; R, reverse primer; SQ, sequencing primer.
