# Supplementary material for: Allelic Expression Imbalance of JAK2 V617F Mutation in BCR-ABL Negative Myeloproliferative Neoplasms
Source: PLoS One. 2013 Jan 22;8(1):e52518. doi: 10.1371/journal.pone.0052518 (PMC3551963; doi:10.1371/journal.pone.0052518)
Supplement: Table S3 — Ratio of cDNA to gDNA of the JAK 2 mutant allelic load in Korean MPN patients. (DOCX) [file pone.0052518.s003.docx]

**Table S3. Ratio of cDNA to gDNA of the *JAK*2 mutant allelic load in Korean MPN patients**

| **MPNs** | **cDNA/gDNA *of JAK2* V617F mutant ratio** | | |
| --- | --- | --- | --- |
|  | **Range** | **Mean^1^** | **SD** |
| PV (n=18) | 1.5-3.2 | 2.2 | 0.6 |
| ET (n=11) | 1.4-5.9 | 3.3 | 1.4 |
| PMF (n=7) | 1.0-1.2 | 1.1 | 0.1 |

^1^Statistically significant difference among each MPNs by Kruskall-Wallis test (see also Figure 2). cDNA, complementary DNA; gDNA, genomic DNA; other Abbreviations as in Table 1.
