# Supplementary material for: Allelic Expression Imbalance of JAK2 V617F Mutation in BCR-ABL Negative Myeloproliferative Neoplasms
Source: PLoS One. 2013 Jan 22;8(1):e52518. doi: 10.1371/journal.pone.0052518 (PMC3551963; doi:10.1371/journal.pone.0052518)
Supplement: Figure S1 — Results of allele-specific PCR with a novel dual-priming oligonucleotide (DPO) system. (DOC) [file pone.0052518.s004.doc]

**
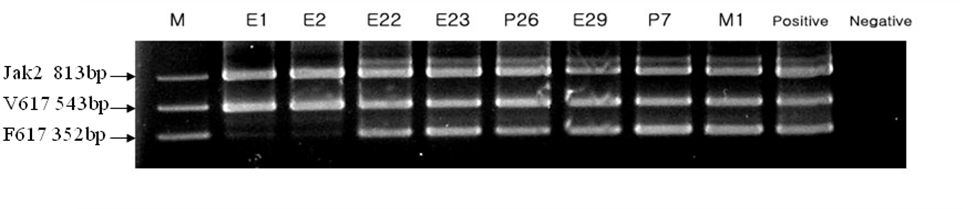
**

| **Sample** | **Result** |
| --- | --- |
| E1 | V617 homo |
| E2 | V617 homo |
| E22 | V617, F617 hetero |
| E23 | V617, F617 hetero |
| P26 | V617, F617 hetero |
| E29 | V617, F617 hetero |
| P7 | V617, F617 hetero |
| M1 | V617, F617 hetero |

**Figure S1. Results of allele-specific PCR with a novel dual-priming oligonucleotide (DPO) system.**
