# Supplementary material for: Allelic Expression Imbalance of JAK2 V617F Mutation in BCR-ABL Negative Myeloproliferative Neoplasms
Source: PLoS One. 2013 Jan 22;8(1):e52518. doi: 10.1371/journal.pone.0052518 (PMC3551963; doi:10.1371/journal.pone.0052518)
Supplement: Figure S2 — Detection of JAK 2 mutation using direct sequencing (A) and REA (B). A: Reverse chromatogram showed wild-type sequence and G to T mutation in JAK2 wild-type pattern in a patient with PMF (left panel). Mixed and mutant sequences in two patients with PV (center and right panel). Arrows indicate the relevant base. B: JAK2 BsaXI digestion were used to genotype DNA from unfractionated peripheral blood leucocytes from patients with ET. (DOCX) [file pone.0052518.s005.docx]

**
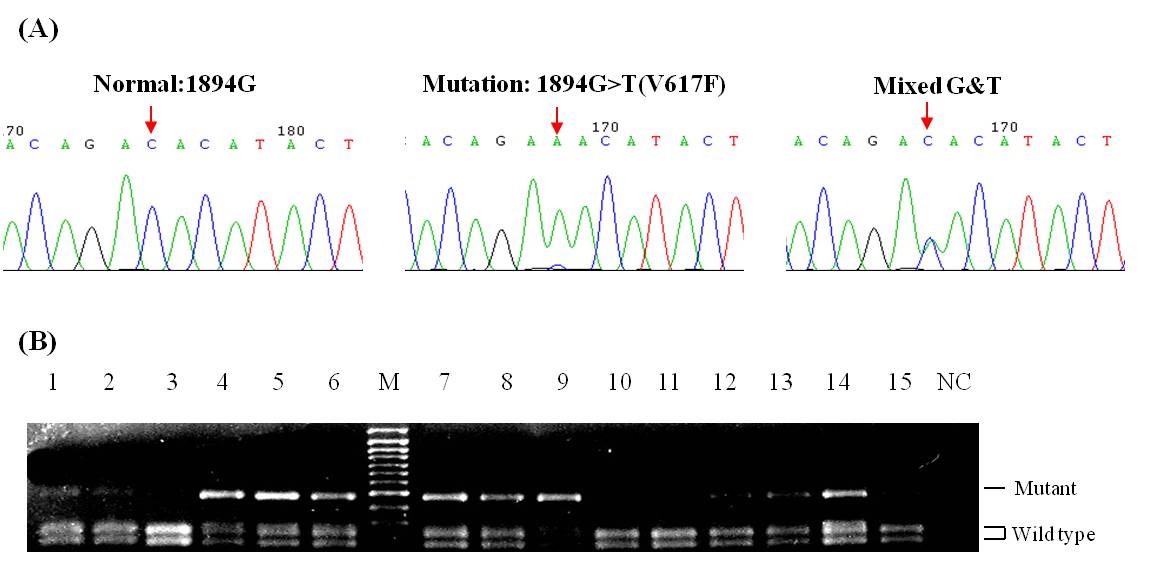
**

**Figure S2. Detection of *JAK*2 mutation using direct sequencing (A) and REA (B).** (A) Reverse chromatogram showed wild-type sequence and G toT mutation in *JAK*2 wild-type pattern in a patient with PMF (left panel). Mixed and mutant sequences in two patients with PV (center and right panel). Arrows indicate the relevant base. (B) *JAK*2 *Bsa*XI digestion were used to genotype DNA from unfractionated peripheral blood leucocytes from patients with ET.
