# Supplementary material for: Allelic Expression Imbalance of JAK2 V617F Mutation in BCR-ABL Negative Myeloproliferative Neoplasms
Source: PLoS One. 2013 Jan 22;8(1):e52518. doi: 10.1371/journal.pone.0052518 (PMC3551963; doi:10.1371/journal.pone.0052518)
Supplement: Figure S3 — Correlation study between quantitative PCR (qPCR) and pyrosequencing (PSQ). Regression analysis of qPCR vs PSQ molecular methods for quantitative determination of JAK2 mutant in the bone marrow cells at diagnosis. Two methods showed good agreement with 0.863 and 0.744 of the correlation coefficient (r) and r-square value (r2), respectively. (DOCX) [file pone.0052518.s006.docx]

**
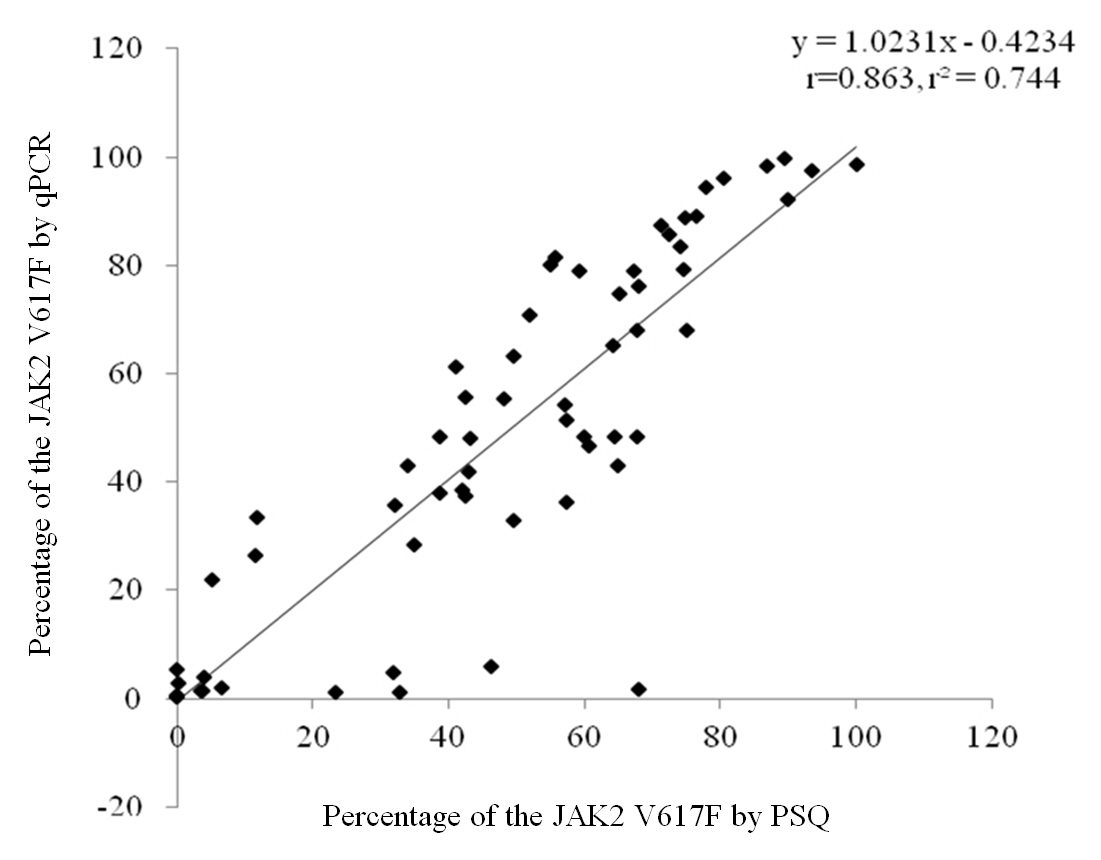
**

**Figure S3. Correlation study between quantitative PCR (qPCR) and pyrosequencing (PSQ).** Regression analysis of qPCR vs PSQ molecular methods for quantitative determination of JAK2 mutant in the bone marrow cells at diagnosis. Two methods showed good agreement with 0.863 and 0.744 of the correlation coefficient (r) and r-square value (r^2^), respectively.
