# Supplementary material for: Allelic Expression Imbalance of JAK2 V617F Mutation in BCR-ABL Negative Myeloproliferative Neoplasms
Source: PLoS One. 2013 Jan 22;8(1):e52518. doi: 10.1371/journal.pone.0052518 (PMC3551963; doi:10.1371/journal.pone.0052518)
Supplement: Figure S4 — TA cloning analysis for further confirmation of heterozygote JAK 2 1849G>T mutation. Direct sequencing chromatogram showed mixtures of JAK2 wild (1849G) and mutant allele (1849T). (DOCX) [file pone.0052518.s007.docx]

**Figure S4. TA cloning analysis for further confirmation of heterozygote JAK2 1849G>T mutation.** Direct sequencing chromatogram showed mixtures of *JAK*2 wild (1849G) and mutant allele (1849T).
