# Supplementary material for: Allelic Expression Imbalance of JAK2 V617F Mutation in BCR-ABL Negative Myeloproliferative Neoplasms
Source: PLoS One. 2013 Jan 22;8(1):e52518. doi: 10.1371/journal.pone.0052518 (PMC3551963; doi:10.1371/journal.pone.0052518)
Supplement: Figure S5 — Scheme of the allele-specific DPO primers for the detection of JAK 2 V617F mutation. Internal control (813 bp) of PCR was used JAK2-F primer and JAK2-R primer. JAK2-F primer and JAK2-wr primer was used for detection of JAK2 wild type (617V, 534 bp). JAK2-R primer and JAK2-mf primer was used for detection of JAK2 mutant type (617F, 352 bp). (DOCX) [file pone.0052518.s008.docx]

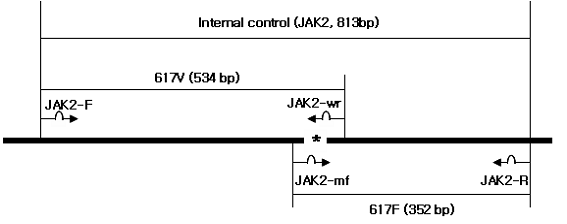


**Figure S5. Scheme of the allele-specific DPO primers for the detection of *JAK*2 V617F mutation.** Internal control (813 bp) of PCR was used *JAK*2-F primer and *JAK*2-R primer. *JAK2*-F primer and *JAK*2-wr primer was used for detection of *JAK*2 wild type (617V, 534 bp). *JAK*2-R primer and *JAK*2-mf primer was used for detection of *JAK*2 mutant type (617F, 352 bp).
